# EUFOREA summit in Brussels 2025: inspiring the future of allergy and respiratory care

**DOI:** 10.3389/falgy.2026.1745834

**Published:** 2026-03-06

**Authors:** S. Lau, V. Backer, G. K. Scadding, P. J. Barnes, M. Bernal Sprekelsen, X. Bertels, M. Blaiss, E. Borzova, M. C. Brüggen, L. O. Cardell, D. M. Conti, M. Cornet, E. De Corso, R. Djukanovic, W. J. Fokkens, A. T. Fox, M. Gaga, P. Gevaert, P. Gibson, C. L. Gray, L. G. Heaney, E. Heffler, H. J. Hoffmann, C. Hopkins, D. Jackson, M. Jesenak, P. Johansen, E. Khaleva, S. Lee, M. J. Mäkelä, E. Melén, J. Mullol, A. Nieto, I. Pavord, A. Peters, D. Price, S. Quirce, D. Ryan, S. Schneider, B. Senior, C. M. E. Shire, P. Smith, T. Teeling, J. C. Virchow, V. Lund, U. Wahn, P. W. Hellings

**Affiliations:** 1 Department of Pediatric Respiratory Medicine, Immunology and Critical Care Medicine, Charité Universitaetsmedizin Berlin, Berlin, Germany; 2 Department of Otorhinolaryngology, Head & Neck Surgery, and Audiology, Rigshospitalet, Copenhagen University, Copenhagen, Denmark; 3 Department of Allergy & Rhinology, Royal National ENT Hospital, London, United Kingdom; 4 Division of Immunity and Infection, University College, London, United Kingdom; 5 National Heart and Lung Institute, Imperial College London, London, United Kingdom; 6 Hospital Clinic, University of Barcelona, Barcelona, Spain; 7 Scientific Expert Team Members, The European Forum for Research and Education in Allergy and Airway Diseases, Brussels, Belgium; 8 Department of Epidemiology, Erasmus MC, Rotterdam, Netherlands; 9 Medical College of Georgia at Augusta University, Augusta, GA, United States; 10 Dermatology Division, Niigata University Graduate School of Medical and Dental Sciences (Medicine), Chuo-ku, Niigata; 11 Department of Dermatology, University Hospital of Zurich, Zurich, Switzerland; 12 Department of Dermatology, University of Zurich, Zurich, Switzerland; 13 Christine Kühne-Center for Allergy Research and Education (CK-CARE), Davos, Switzerland; 14 ToxiTEN Group, European Reference Network for Rare Skin Diseases (ERN-Skin), Paris, France; 15 Division of ENT Diseases, Department of Clinical Sciences, Intervention and Technology, Karolinska Institutet, Stockholm, Sweden; 16 Department of ENT Diseases, Karolinska University Hospital, Stockholm, Sweden; 17 Allergy and Clinical Immunology Research Unit, Department of Microbiology and Immunology, KU Leuven, Leuven, Belgium; 18 Escuela de Doctorado UAM, Centro de Estudios de Posgrado, Universidad Autónoma de Madrid, Calle Francisco Tomás y Valiente, n° 2. Ciudad Universitaria de Cantoblanco, Madrid, Spain; 19 Department of Otorhinolaryngology, Alrijne Hospital, Leiderdorp, Netherlands; 20 Otolaryngology Head and Neck Surgery, A. Gemelli University Hospital Foundation IRCCS, Rome, Italy; 21 Faculty of Medicine, University of Southampton, Southampton, United Kingdom; 22 Department of Otorhinolaryngology, Amsterdam University Medical Centres, Amsterdam, Netherlands; 23 Children’s Allergy Service, Evelina London Children’s Hospital, London, United Kingdom; 24 Department of Paediatric Allergy, King’s College London, London, United Kingdom; 25 Section of Pulmonary and Critical Care Medicine, Hygeia Hospital, Athens, Greece; 26 Upper Respiratory Diseases Research Group, University of Ghent, Gent, Belgium; 27 Department of Respiratory and Sleep Medicine, John Hunter Hospital, Newcastle, NSW, Australia; 28 Priority Research Centre for Healthy Lungs, College of Health, Medicine and Wellbeing, University of Newcastle, Hunter Medical Research Institute, Newcastle, NSW, Australia; 29 Division of Allergy, Department of Paediatrics and Child Health, University of Cape Town, Rondebosch, South Africa; 30 KidsAllergy Centre, Cape Town, South Africa; 31 Wellcome-Wolfson Institute for Experimental Medicine, Belfast, United Kingdom; 32 IRCCS Humanitas Research Hospital, Rozzano, Italy; 33 Humanitas University, Pieve Emanuele, Italy; 34 Department of Clinical Medicine, University of Aarhus, Aarhus, Denmark; 35 Department of Rhinology and Skull Base Surgery, Guy’s and St Thomas’ Hospital NHS Foundation Trust, London, United Kingdom; 36 Guy’s Severe Asthma Centre, Guy’s and St. Thomas’ NHS Foundation Trust, and the School of Immunology and Microbial Sciences, King’s College London, London, United Kingdom; 37 Department of Pulmonology and Phthisiology, Department of Pediatrics, Department of Clinical Immunology and Allergology, Jessenius Faculty of Medicine in Martin, Comenius University in Bratislava, University Hospital in Martin, Martin, Slovakia; 38 Clinical and Experimental Sciences and Human Development and Health, Faculty of Medicine, University of Southampton, Southampton, United Kingdom; 39 NIHR Southampton Biomedical Research Centre, University Hospital Southampton NHS Foundation Trust, Southampton, United Kingdom; 40 Division of Rhinology and Skull Base Surgery, Department of Otolaryngology-Head and Neck Surgery, Johns Hopkins School of Medicine, Baltimore, MD, United States; 41 HUS Skin and Allergy Hospital, University of Helsinki and Helsinki University Hospital, Helsinki, Finland; 42 Department of Clinical Science and Education Södersjukhuset, Karolinska Institutet and Sachs’ Children and Youth Hospital, Stockholm, Sweden; 43 Rhinology Unit and Smell Clinic, ENT Department, Hospital Clínic, IDIBAPS, Universitat de Barcelona, CIBERES, Barcelona, Spain; 44 Health Research Institute, Hospital La Fe, Valencia, Spain; 45 Respiratory Medicine, NIHR Oxford Biomedical Research Centre, Nuffield Department of Medicine, University of Oxford, Oxford, United Kingdom; 46 Department of Medicine, Division of Allergy and Immunology, Northwestern University Feinberg School of Medicine, Chicago, IL, United States; 47 Observational and Pragmatic Research Institute, Singapore, Singapore; 48 Optimum Patient Care Global, Cambridge, United Kingdom; 49 Centre of Academic Primary Care, Division of Applied Health Sciences, University of Aberdeen, Aberdeen, United Kingdom; 50 Department of Allergy, Hospital Universitario La Paz, IdiPAZ, Madrid, Spain; 51 Allergy and Respiratory Research Group, Usher Institute of Population Health Sciences and Informatics, University of Edinburgh, Edinburgh, United Kingdom; 52 International Primary Care Respiratory Group, Edinburgh, United Kingdom; 53 Department of Otorhinolaryngology, Head and Neck Surgery, Vienna General Hospital, Medical University of Vienna, Vienna, Austria; 54 Department of Otolaryngology/Head and Neck Surgery, University of North Carolina, Chapel Hill, NC, United States; 55 Patient Advisory Board of the European Forum for Research and Education in Allergy and Airway Diseases, Brussels, Belgium; 56 Griffth University, Southport, QLD, Australia; 57 Klinik für Pneumologie, Allergologie und Internistische Intensivmedizin, Universitätsmedizin Rostock, Rostock, Germany; 58 Royal National Ear, Nose and Eastman Dental Hospital, London, United Kingdom; 59 Royal National Ear, Nose and Throat and Eastman Dental Hospitals, University College London Hospitals, London, United Kingdom; 60 Department of Pediatric Respiratory Medicine, Immunology and Critical Care Medicine, Charité Universitaetsmedizin Berlin, Berlin, Germany; 61 Department of Otorhinolaryngology-Head and Neck Surgery, University Hospitals Leuven, Leuven, Belgium

**Keywords:** allergen immunotherapy, asthma, COPD, EUFOREA, paediatric allergy, rhinitis, rhinosinusitis, summit

## Abstract

EUFOREA, the European Forum for Research and Education in Allergy and Airways diseases, is an international non-for-profit organisation of physicians, patients, and other stakeholders committed to the improvement of outcomes for patients suffering from chronic inflammatory respiratory conditions through collaborative research, education, and targeted advocacy. In February 2025, EUFOREA hosted its biennial summit in Brussels, convening expert panels, its Patient Advisory Board, and a range of leadership representatives. This event was pivotal in establishing the research, educational, and advocacy priorities for the subsequent 2-year period, coinciding with the organisation's 10th anniversary in April 2025. Building on its core competencies, EUFOREA advances evidence-based clinical tools and educational initiatives designed to narrow the gap between emerging science and routine care. In alignment with the objective of enhancing global healthcare standards, the specialists' panels of chronic rhinosinusitis and European Position Paper on Rhinosinusitis and Nasal Polyps (EPOS), allergic rhinitis, asthma, allergen immunotherapy, and paediatrics have devised and detailed a comprehensive range of initiatives that address significant unmet needs within the domains of allergy and respiratory care. This document presents a summary of EUFOREA's future objectives, aspirations, and strategy, providing a clear direction for the coming years. It serves as a valuable resource for individuals and organisations involved in both the allergy and the respiratory sectors, offering a comprehensive overview of the association's plans and initiatives.

## Introduction

Chronic respiratory diseases (CRDs), including asthma, allergic rhinitis (AR), chronic rhinosinusitis (CRS), and chronic obstructive pulmonary disease (COPD), constitute a growing public health burden worldwide, with substantial consequences for morbidity, health-related quality of life, and socio-economic cost (1–4). These conditions are characterised by overlapping pathophysiology and frequent comorbidity across the upper and lower airways. However, clinical care and guidelines have historically focused on organ-specific problems in relative isolation. This fragmentation has the effect of limiting opportunities for integrated prevention strategies, early detection of progressive disease, and coordinated multidisciplinary management. To address these gaps, EUFOREA operates as a multidisciplinary platform that integrates clinical expertise, patient perspectives, and education with a focus on implementation to accelerate the translation of evidence into practice (5–9).

A number of challenges have been identified as hindering the efforts to enhance prevention and improve the quality of care, and some requirements have also been suggested (1): the absence of effective cooperation amongst healthcare organisations, the division of the respiratory tract between disciplines, the lack of emphasis on preventive measures and excellence in care, a need for improved cooperation amongst other stakeholders, a noticeable absence of integrated advocacy efforts to raise awareness of the burden of disease around the world, a necessity for a genuinely international patient advisory board, comprising individuals affected by CRDs, to articulate a unified perspective, and the lack of smart use of available big health data. The aforementioned issues are in addition to those that specifically affect patients, such as inequalities of care and limited empowerment to face a medical consultation.

Funded on the basis of its primary commitment to inclusivity and scientific rigour, EUFOREA functions as a convening platform for innovation and education across the healthcare continuum, with the ultimate goal of optimising patient outcomes. EUFOREA embodies a multidisciplinary spirit, bringing together primary care physicians, pulmonologists, allergists, ENT surgeons, paediatricians, and patients. The organisation employs an inclusive framework to proactively encourage innovation in the diagnosis, treatment, and long-term management of CRDs. In addition, it engages in advocacy across policy and regulatory domains to ensure that patient-centred perspectives inform respiratory-health decision-making.

**Figure 1:**
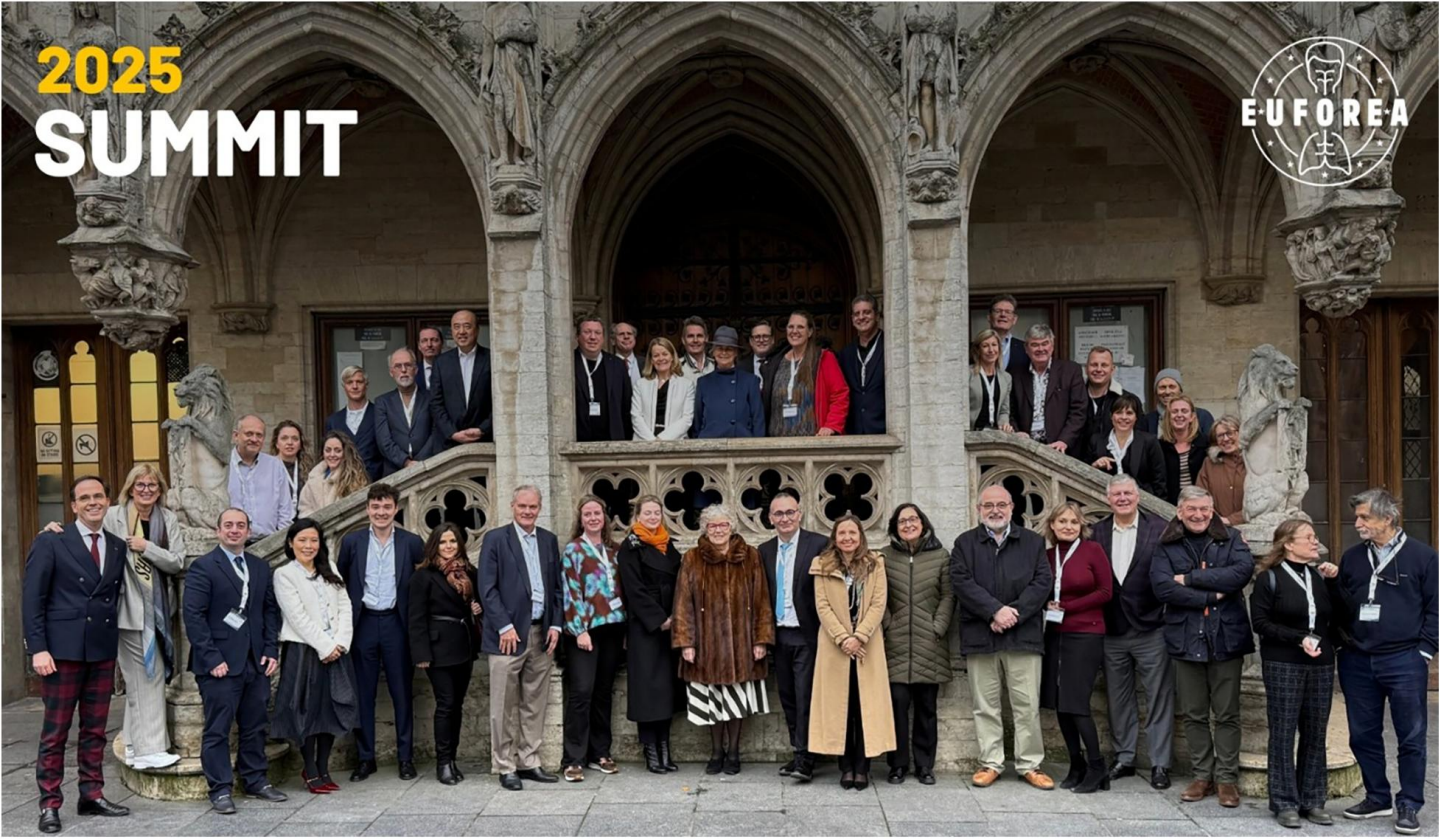
Photograph captured on the occasion of the 2025 EUFOREA summit hosted in Brussels. Photograph by Diego Conti, 2025.

In alignment with this mission, the 2025 EUFOREA summit convened members of the patient advisory board (PAB) alongside expert panels in asthma, AR, CRS, and paediatrics to collectively identify and prioritise activities for the coming biennium (Figure 1). Since its foundation, EUFOREA has achieved numerous milestones, which are detailed on its website under the banner “inspiring the future of respiratory care” (https://www.euforea.eu) As illustrated in Table 1, this report offers a concise synthesis of accomplishments, future aspirations, and the proposed action plan. Stakeholders from across the allergy and respiratory spectrum were informed and invited to collaborate in addressing the substantial burden of preventable CRDs both within Europe and beyond. The discussions held during the course of the summit were structured around the unmet needs identified by each expert panel; proposals were evaluated in terms of their novelty, alignment with EUFOREA's mission and values, and potential impact. There was a unanimous recognition amongst the co-authors of the critical importance of stakeholder collaboration [including patients (10), clinicians, researchers, policymakers, and industry partners] in driving forward meaningful, system-level changes.

**Table 1:** Overview of the achievements of EUFOREA.

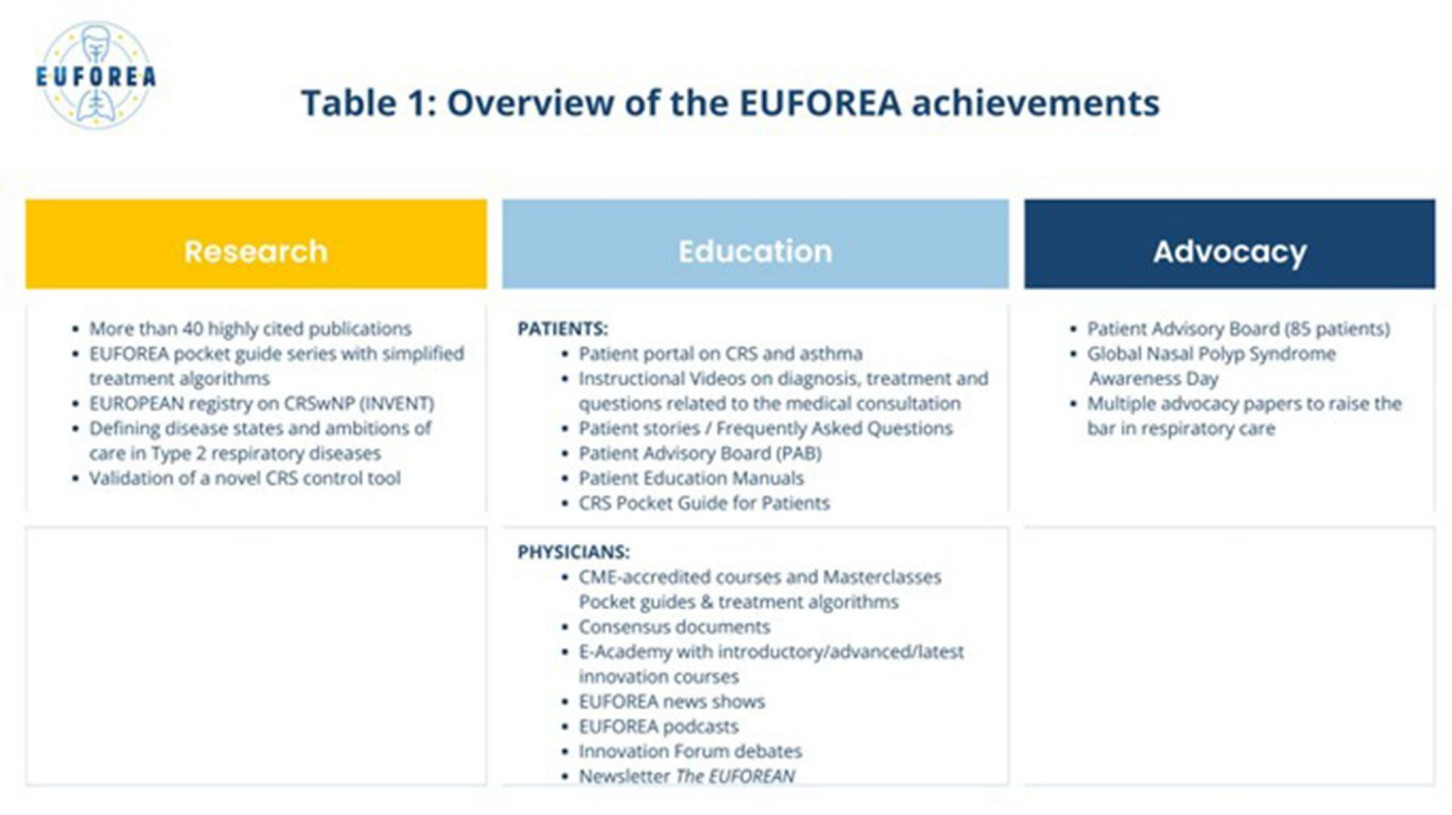

## Research

The expert panels of EUFOREA have emphasised the importance of collaborative work, which has led to the publication of multiple papers since its foundation. EUFOREA has contributed to optimal care through the development and dissemination of pocket guides (PGs) with simplified algorithms on AR for adults (11) and for children (12), on CRS (13) and, more recently, on asthma (14) and on biologics for Type 2 respiratory diseases (15). These physician-oriented guides are complemented by patient guides in lay language (16), which have been endorsed and positively received by the Patients Advisory Board and overall patient community worldwide.

Building further on unmet needs in the field, the EUFOREA experts will develop the following PGs with simplified treatment guidance and algorithms for paediatric asthma, COPD, global type 2 inflammation PG, and for parents/patients with paediatric asthma. Aligned with these ambitions, a decision has been taken to proceed with multiple translations of several PGs to make these clinical guides accessible to all those for whom they may be beneficial to understand optimal care pathways.

In addition to the research-related initiatives that have already been adopted in clinical practice, such as the definition of disease states in allergic rhinitis (17), chronic rhinosinusitis (18), and asthma (14), EUFOREA will launch a new tool to assess self-perceived control and severity in patients with CRS, called the CRS control test (CCT). Once validated and launched, this CCT will be articulated with a screening and referral tool for upper and lower airways and a global airway disease control test, which was identified as an important unmet need and underlines the multidisciplinary ambitions of EUFOREA. The initiative to develop a EUFOREA control test (ECT) is grounded in the understanding that asthma, COPD, CRS, and AR are highly prevalent and interconnected respiratory conditions. However, clinical practice and trial frameworks continue to treat these conditions in isolation, with a neglect of symptoms at the other end of the respiratory tract. The lack of a unified tool to measure control across upper and lower airway diseases is a critical unmet need. With this in mind, EUFOREA will develop and validate a short, clinically practical control instrument that quantifies control in both the upper and the lower respiratory tract and could also be used in research to document treatment outcomes with a more patient-relevant focus.

While there is evidence to suggest that biologics are effective treatment options for chronic rhinosinusitis with nasal polyps (CRSwNP) (19, 20), asthma (15, 21), and eosinophilic COPD (22), there are significant gaps in knowledge and unanswered critical questions regarding clinical use. Given the complexity of the mechanisms of action of biologics in CRDs, the potential for undesired outcomes remains significant. The identification of optimal patient selection criteria for biologic therapy or surgical interventions, including the determination of patients best placed to benefit, remains a field of continuous investigation. Currently, many researchers are actively involved in studies on predictive biomarkers. In order to agree on major clinical strategies of care, EUFOREA will organise two consensus meetings per year on topics of relevance, such as remission in CRS and asthma, surgery and/or biologics for CRSwNP, criteria of the choice of a biologic, strategies for prevention, and other topics of relevance based on the dynamic evolution in the respiratory field. The role of newly approved drug therapies and biologics will also be discussed. Consensus meetings feature the participation of global experts in the respiratory field, patients, and strategic partners of EUFOREA.

The EUFOREA registry on real-world efficacy of biologics for the indication of CRSwNP (INVENT) is a pioneering international initiative designed to transform CRSwNP care based on combined insights from large national registries. By unifying real-world clinical data from across different countries, INVENT aims to evaluate the effectiveness of biologic therapies in everyday practice, with vital insights for clinicians, payers, and regulators. In the landscape of growing numbers of available biologics, the optimal choice and timing of biologic therapy remain uncertain. While EUFOREA has contributed to the field with its pocket guide on biologics (15), updating the content is warranted on an annual basis given the dynamics in the field.

## Education

Education of both physicians and patients is one of the fundamental pillars of EUFOREA, providing both parties with simple, comprehensive, and relevant information and knowledge to improve healthcare outcomes. To date, over 20,000 physicians have participated in accredited (online and physical) CME courses, or masterclasses offered by EUFOREA over the past 5 years, while instructional/educational videos for patients have garnered over 1 million online views. The importance of easily accessible educational materials was highlighted by expert panels. The development of credible educational material for physicians in the e-Academy and for patients in the Patient Educational Portal of EUFOREA should prove to be important tools to improve outcomes of care.

Following up on the success of the CME-accredited European Biologic Training Courses (EBTC) by EUFOREA, this initiative will continue on a yearly basis with the 2026 edition organised in the Royal Society of Medicine in London. The following aims of the EBTC by EUFOREA have been agreed upon: (1) gain an understanding of biologic use in asthma, COPD, and CRSwNP, (2) navigate the selection, initiation, and follow-up of biologic treatment, (3) learn to evaluate treatment outcomes and adjust strategies accordingly, and (4) enhance collaboration across disciplines for integrated, patient-centred care.

The EUFOREA e-Academy (23) is an advanced digital learning platform developed to support the ongoing education and professional development of healthcare providers in the field of chronic inflammatory respiratory disease. Designed to meet the highest standards in clinical education, the platform offers a comprehensive curriculum of CME-accredited courses accessible globally and on demand, with different levels of knowledge and expertise, from introductory level for GPs, specialists-in-training, to latest innovations for specialists-in-practice. The range of courses encompasses training in AR, CRS, asthma, and allergen immunotherapy (AIT), with levels ranging from introductory to advanced and including the latest innovations. This e-Academy also includes EUFOREA's CME-accredited masterclasses addressing pivotal and evolving themes in the management of airway diseases, hence serving as a platform for scientific advancement, clinical education, and strategic dialogue amongst healthcare professionals and thought leaders. Each Masterclass focuses on a central, high-impact topic, with recent programmes exploring the transformative role of biologic therapies in the treatment of asthma and CRS. The emergence of these therapies has redefined clinical expectations, shifting the paradigm from disease control towards the ambitious goals of remission and, increasingly, potential cure—as reflected in the most recent international treatment guidelines. A masterclass on real-world efficacy of biologics, a masterclass on remission in asthma and CRSwNP, and a masterclass on novel approaches for severe AR will also be available soon. The following educational activities are planned for physicians in 2025–2027 in the context of PGs and E-Academy development: COPD, biomarkers in Type 2 inflammation and updates on rapidly evolving fields like biologics for type 2 inflammation, and strategies for secondary and tertiary prevention.

The most significant unmet need expressed by patients of the PAB of EUFOREA has been a patient educational portal for patients with asthma, COPD, AR, and Nasal Polyp Syndrome (or CRSwNP) (24). In this context, there is a gradual launch of modules foreseen in 2025 and 2026, addressing the unmet patient needs, which represents the first stage of the Airways Disease Action Plan for Predictive and Preventive treatment (ADAP^3^T). This educational material will be included in digital tools supporting and empowering patients with type 2 inflammatory conditions of the respiratory tract.

Finally, the EUFOREA Innovation Forum is a unique and dynamic platform designed to foster open, balanced, and forward-looking dialogue on the future of innovation in respiratory care. Broadcast from EUFOREA's state-of-the-art augmented reality studio, these highly engaging sessions bring together a diverse panel of stakeholders for in-depth discussions on the scientific, clinical, economic, and policy dimensions of transformative respiratory therapies. Each forum is moderated by a professional medical journalist and features multidisciplinary voices—leading clinicians, academic researchers, patients, payers, and industry representatives—ensuring a rich, multi-perspective debate on key issues shaping the respiratory health ecosystem. The Innovation Forum is more than a discussion series—it is a strategic vehicle for stakeholder alignment, knowledge dissemination, and market shaping. Through its ability to illuminate unmet needs, explore implementation barriers, and highlight best practices, the Innovation Forum directly supports the acceleration of innovative treatment pathways and healthcare. In order to ensure the dissemination of accurate health-related information, EUFOREA will continue to communicate and bring up-to-date health content not only through traditional academic channels, but also through social media and its well-known EUFOREA news shows, Innovation forum debates, and its newspaper, The EUFOREAN. In addition to these, the EUFOREA On Air podcast series has been launched, which is complemented by the podcast series “Polyp Gossip,” thus covering the range of options and providing a closer contact.

## Advocacy

Advocacy is a pivotal part of driving beneficial transformation in society and tackling challenges that impact the individual and collective (7). Despite the epidemic proportions of respiratory conditions such as asthma, CRS, COPD, and AR, many individuals remain unaware of their disorder, hence remaining undiagnosed and untreated. Moreover, there is a lack of awareness of the impact of under- or misdiagnosis on the missed opportunity for prevention.

The course of CRDs is often characterised by a natural history of steady progress of the disease with exacerbation in severity and the acquisition of comorbidities (25). It is therefore essential to raise awareness of CRDs amongst patients and healthcare providers to ensure that people experiencing symptoms receive early and accurate diagnosis and are being offered an appropriate strategy for prevention and optimal management. In addressing the unmet needs of CRSwNP patients, EUFOREA organises a yearly World Nasal Polyp Syndrome Awareness Day with endorsement of a growing number of international organisations (5). Building on these efforts, EUFOREA organised a global symposium on Raising the bar in CRDs in the European Parliament in 2024 (26) and will continue to organise advocacy events including a respiratory summit at the Royal Society of Medicine in London in June 2026.

Although the use of patient-reported outcome measures (PROMs) is being adopted more widely, there has been little focus on how patients perceive clinically defined outcomes. Therefore, EUFOREA will launch a Patient Survey on Disease States and Therapeutic Goals to investigate the understanding and knowledge of patients about their treatment goals. This project will provide insights into how EUFOREA can help patients and physicians alike to define and communicate on treatment goals and ambitions of care.

It is imperative to acknowledge the significance of raising awareness not only amongst the general public but also amongst healthcare decision-makers. EUFOREA encourages and organises meetings with the 75 patients of the PAB. The active engagement of individuals afflicted by these conditions is essential to understanding their individual circumstances and the impact they have on their lives. In this regard, EUFOREA has published several papers that capture the patient's perspective on pathways of care and suggestions to overcome the shortcomings of current care pathways (5–7). In addition, EUFOREA, in partnership with a leading health organisation, will convene a respiratory summit—a closed-door, high-level meeting in June 2026. The summit will gather global leaders from guidelines committees, clinical societies, patient organisations, and industry and policy to discuss the implementation of optimal respiratory care. Despite the increased accessibility of essential as well as innovative medicines such as biologics and their established role in the treatment of chronic respiratory diseases, their availability remains limited and dissimilar around the world. There are still many significant challenges in providing optimal treatment to patients globally. In this context, EUFOREA is undertaking a global study (as part of the INVENT initiative) to investigate product availability, regulatory standards, and costs. The objective of this exercise is to underscore these discrepancies and stimulate a discourse on the substantial indirect expenses associated with respiratory tract diseases, which frequently remain unacknowledged and can be substantially mitigated by readily available treatment.

Patient education for CRDs has been identified as an under-addressed means of empowering patients, and EUFOREA has an important role to play in improving the knowledge of both patients and healthcare professionals. A better understanding of their condition enables patients to advocate for better care in their local communities. EUFOREA proposes an ambitious training course (Patient Experts in Airways and Respiratory Leadership; PEARL) designed to train patients to become experts of their condition and aimed at empowering them. The core values of this PEARL course are the following:

1. educate international CRD patients on disease mechanisms and evidence-based care,
2. elevate the patient voice in healthcare systems and clinical decision-making,
3. empower patients to represent the patient voice at national reimbursement committees, and
4. establish a global network of certified patient experts under the EUFOREA umbrella.

A summary of the activities for the 2025–2027 period in the fields of different expert panels is provided in Table 2.

**Table 2:** Activities for the 2025–2027 period in the fields of different expert panels.

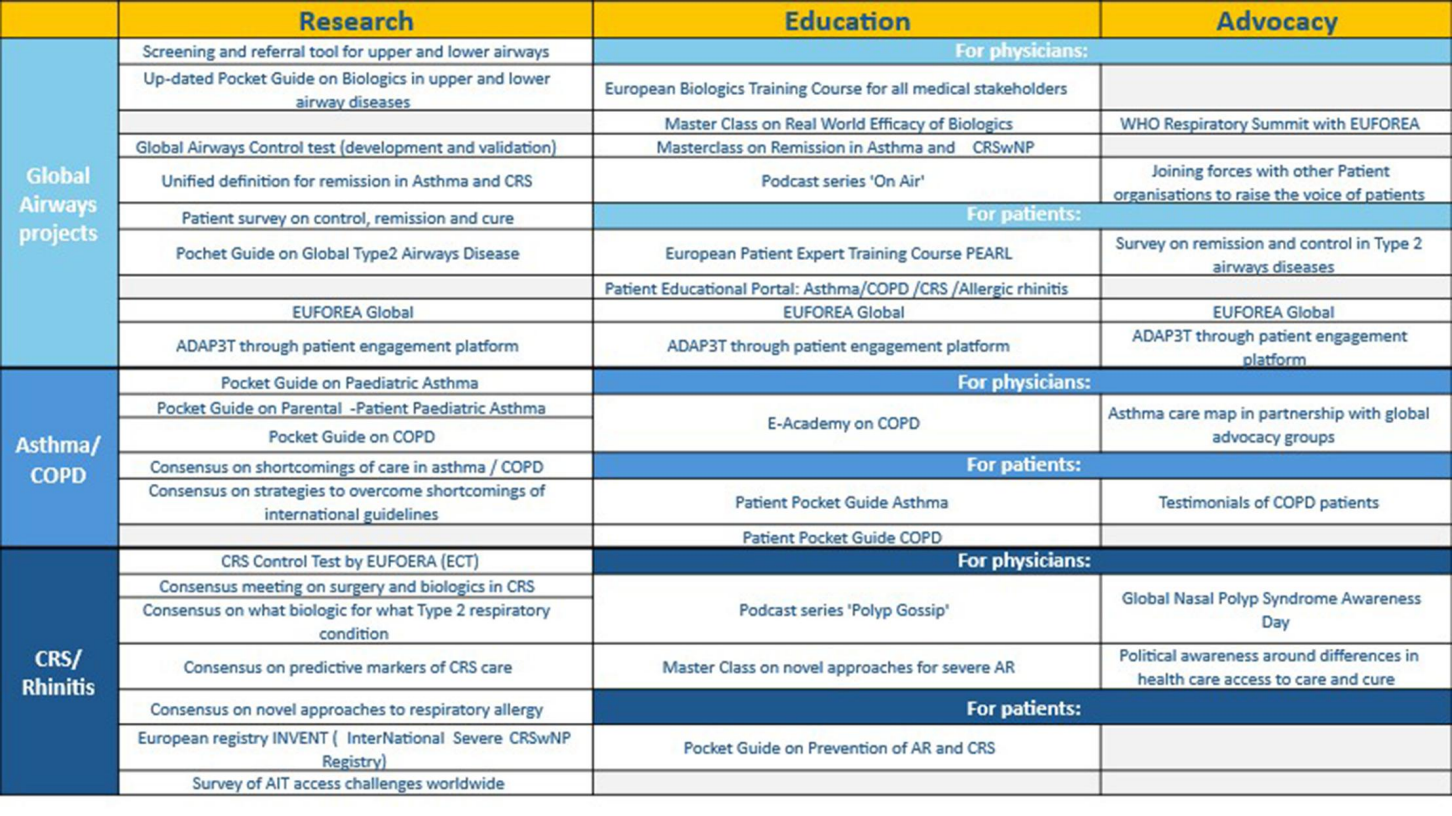

## Summary

In order to address the rising prevalence and societal impact of chronic inflammatory airway diseases, there is a necessity for coordinated, multidisciplinary efforts across research, education, and policy. The strategy devised by EUFOREA seeks to expedite the implementation of preventive and precision approaches, reduce avoidable disparities in care, and promote equitable access to effective interventions. This strategy is founded upon the integration of concise clinical tools, scalable educational initiatives, and targeted advocacy efforts.

The 2025 summit articulated a set of priorities that establish a pragmatic roadmap for advancing integrated airway care in Europe and beyond. The success of this initiative is reliant on the sustained collaboration amongst clinicians, patients, researchers, industry partners, and policy makers beyond national borders and different healthcare systems. EUFOREA remains committed to its mission of encouraging all parties to take action in implementing advocacy and personalised medicine as effective tools to address the challenge of CRDs.

## Data Availability

The original contributions presented in the study are included in the article/supplementary material, and further inquiries can be directed to the corresponding author.
